# Class enumeration false positive in skew-*t* family of continuous growth mixture models

**DOI:** 10.1371/journal.pone.0231525

**Published:** 2020-04-17

**Authors:** Kiero Guerra-Peña, Zoilo Emilio García-Batista, Sarah Depaoli, Luis Eduardo Garrido

**Affiliations:** 1 School of Psychology, Pontificia Universidad Católica Madre y Maestra, Santiago de los Caballeros, Dominican Republic; 2 Department of Psychology, University of California, Merced, California, United States of America; Roswell Park Cancer Institute, UNITED STATES

## Abstract

Growth Mixture Modeling (GMM) has gained great popularity in the last decades as a methodology for longitudinal data analysis. The usual assumption of normally distributed repeated measures has been shown as problematic in real-life data applications. Namely, performing normal GMM on data that is even slightly skewed can lead to an over selection of the number of latent classes. In order to ameliorate this unwanted result, GMM based on the skew *t* family of continuous distributions has been proposed. This family of distributions includes the normal, skew normal, *t*, and skew *t*. This simulation study aims to determine the efficiency of selecting the “true” number of latent groups in GMM based on the skew *t* family of continuous distributions, using fit indices and likelihood ratio tests. Results show that the skew *t* GMM was the only model considered that showed fit indices and LRT false positive rates under the 0.05 cutoff value across sample sizes and for normal, and skewed and kurtic data. Simulation results are corroborated by a real educational data application example. These findings favor the development of practical guides of the benefits and risks of using the GMM based on this family of distributions.

## Introduction

Growth Mixture Modeling (GMM) has gained great popularity in the social sciences as a group of longitudinal data modeling techniques for the selection of the number of latent trajectory classes, and the estimation of the specific relation between these classes and level 1 or level 2 covariates [1–5]. These trajectories, or growth curves, could be interpreted as substantively different groups of patients suffering from major depression according to their response to antidepressants [6], adolescents’ developmental trajectories of Anxiety [7], groups with quantitatively different alcohol use trajectories [8], or different clinical groups of alcohol abuse patients [9].

Worth noting is that GMM, with normally distributed random errors, is generally used for two purposes: 1) to identify mixtures of normal sub-groups within a sample of a population, and 2) to better approximate oddly shaped probability densities by a mixture of normally distributed components [10]. Only in the first case is assigning substantive meaning to latent classes appropriate. However, the same fit statistics and likelihood ratios test (LRT) are used to select the number of latent components in both applications. This represents the biggest challenge in GMM: distinguishing between latent classes that represent substantively different groups from those necessary to approximate an unusually shaped density. For example, a researcher fitting a normal GMM to a population with a single group with a lognormal distribution will most likely over select the number of latent classes, since at least one more latent component will be needed to approximate the right heavy tail of the lognormal distribution [10].

Situations in which spurious latent classes can be selected were pointed out previously in the literature [10–12], and can be summarized as violations of the assumptions of the fitted model. The normality assumption is of special interest since real world data in social sciences is rarely normal [13]. Moreover, Guerra-Peña and Steinley [14] point out the limitations of using fit indices and LRT to correctly select the number of latent trajectory classes in normal GMM, even when the skewness and/or kurtosis are only present in the covariates and not the repeated measures. In order to deal with this issue, continuous nonnormal skewed distributions have been developed [15] and extended onto GMM [16]. These authors specifically focused on the restricted multivariate skew *t* distribution. This restricted case of the skew *t* family of continuous GMMs is readily available in the statistical package Mplus 7.1 [17]. Nevertheless, the effectiveness of using fit indices and LRT tests to solve the mentioned limitation in the skew *t* family of continuous distributions has not been studied comprehensively.

The purpose of this research is to do so simulating data conditions more representative of those found in social science research. This paper is intended to guide the applied researcher in the advantages and limitations of this analysis, thus the restricted skew *t* GMM was used here for being the parametrization most readily available in statistical packages, namely Mplus. The rest of this paper is organized as follows: first we give a brief overview of normal GMM and its well documented classification problems, then we do the same with nonnormal skewed continuous GMMs, we continue by stating the goals and design of this simulation study, we present simulation and real data example results, and finally, we discuss relevant findings.

### Normal growth mixture modeling

Normal GMM is useful when you have longitudinal data, such as antisocial behavior test scores over the life span of a group [18] or patterns of alcohol use from teenage to adult years [8], and you wish to identify subgroups or latent classes with different development patterns. GMM helps identify these subgroups in the absence of a classification variable by testing whether the data derive from a mixture of populations. The principal aim here is to classify each individual in one of these latent classes while minimizing selection error.

Traditional GMM has the underlying assumptions that the repeated measures follow the normal distribution, thus the latent classes were a mixture of normal components. GMM is commonly known as mixture modeling and it makes use of full information estimation and mixed models [2, 19–21].

Following the notation of Asparouhov and Muthén [16] the general equations in matrix form for GMM are

$$\begin{array}{c} Y_{i}=\nu^{\left(g\right)}+\Lambda^{\left(g\right)}\eta_{i}^{\left(g\right)}+\epsilon_{i}^{\left(g\right)} \end{array}$$ (1)

$$\begin{array}{c} \eta_{i}^{\left(g\right)}=\alpha^{\left(g\right)}+B_{\eta_{i}}^{\left(g\right)}+\Gamma^{\left(g\right)}X_{i}^{\left(g\right)}+\xi_{i}^{\left(g\right)}, \end{array}$$ (2)

where

$$\begin{array}{c} \left(\epsilon_{i}^{\left(g\right)},\xi_{i}^{\left(g\right)}\right)∼MN\;\left(0,\Sigma_{0}^{\left(g\right)}\right), \end{array}$$ (3)

and

$$\begin{array}{c} \Sigma_{0}^{\left(g\right)}=(\begin{array}{cc} \Theta^{\left(g\right)} & 0\\ 0 & \Psi^{\left(g\right)} \end{array}) \end{array}$$ (4)

In Eq (1) we have that ***Y***_**i**_ is a vector of repeated measures, ***ν***^(*g*)^ is a vector of level 1 intercepts, **Λ**^(*g*)^ is a matrix of factor loadings, $\eta_{i}^{\left(g\right)}$ is a vector of *m* latent factors, and ***ϵ***^(*g*)^ is a vector of level 1 random errors. In Eq (2), ***α***^(*g*)^ is a vector of level 2 intercepts (factor means), $B_{\eta_{i}}^{\left(g\right)}$ is a vector of random slopes for the latent factors, **Γ**^(*g*)^ is a matrix of random slopes of the repeated measures on the covariates **X**^(*g*)^, and $\xi_{i}^{\left(g\right)}$ is a vector of level 2 random errors. The *g* superscript in Eqs (1)–(4) indicates that each latent class has a different set of these terms. As Eq (3) shows, $\epsilon_{i}^{\left(g\right)}$ and $\xi_{i}^{\left(g\right)}$ are normally distributed in this formulation. It is important to note that there are other mixture models for longitudinal data analysis not mentioned in the present study. For an example, see the model proposed in Ng et al. [22].

### Classification problems of normal GMM

Since its development, normal GMM has been proven to have several problems. These problematic issues concern, but are not limited to, the maximum likelihood estimators (MLEs) yielding non-consistent estimates under violations of the multivariate normality assumption [21]; multiple latent class trajectories can be selected and appear optimal even when only one group exists in the data [10, 11]; spurious latent classes can be estimated when the structural model is misspecified and when the relationship among observed and/or latent variables is nonlinear [12]; a large number of random starts are needed to avoid local optimal solutions [23] and non-convergence issues [24, 25].

Concerning the use of fit indices in the selection of the number of latent classes, the Akaike information criterion (AIC) overestimates the number of latent components even when the repeated measures are normally distributed [26–28], and when the data are nonnormal [10–12, 14]. The Bayesian information criterion (BIC) underestimates the number of groups compared to the “true” model when sample size is small [27, 29], overestimates the number of latent classes with nonnormal data and large sample sizes [14], and both the BIC and the sample corrected BIC (SBIC) overestimate the number of latent classes when the model has been misspecified [12] or the data are nonnormal [10–12, 14]. Moreover, fit indices can distinguish between classes with different trajectories only if they are well separated and as sample size increases [29].

Bauer and Curran [12] give three conditions in which fit indices favor solutions with spurious latent classes: nonnormal continuous observations, model misspecification, and nonlinear relationships between latent and/or observed variables. In these cases, these authors point out the likelihood function for the single class solution is a poor representation of the data and the likelihood function of a “false” multiple-group solution offers a better fit [10].

Likelihood ratio tests (LRT) compare the likelihood functions of a particular k-class solution with the k-1-class results. The test statistic provided indicates which solution fits the data better. Since fit indices for competing models cannot be compared by a test statistic (e.g., only selecting the model with the smaller fit index within a group of nested models), LRTs have been readily developed as an alternative to using fit indices to make the decision about the number of latent classes in the data [30, 31]. The Voung-Lo-Mendel-Rubin likelihood ratio test (VLMR-LRT) [32] has been proven problematic [33]. Regardless, some authors suggest that it might still be useful for class enumeration [32, 34]. Moreover, the Lo-Mendel-Rubin adjusted likelihood ratio test (LMR-adjusted LRT) has been corrected for the VLMR-LRT problem [32].

The bootstrap likelihood ratio test (BLRT) [28] is a parametric bootstrap method for comparing nested models in GMM. Jung and Wickrama [35] conducted a simulation study with nonnormal observer variables. The authors considered skewness and kurtosis values of 1 on the repeated measures and found that BLRT performed best among likelihood ratio tests and fit indices, except for the BIC and SBIC. Guerra-Peña and Steinley [14] showed that the BLRT performs better than other LRT for both normal and nonnormal repeated measures. Nevertheless, even in normal conditions, type I error rates where 5% or higher, becoming worse as sample size increases (e.g., *N* = 800) and the ratio of kurtosis to skewness becomes larger (e.g., skewness of 0 and kurtosis of 4).

### GMM with continuous nonnormal skewed distributions

Azzalini [36] first proposed the skew normal distribution and Azzalini and Dalla Valle [37] extended it to the multivariate skew normal distribution. The addition of skewness parameters to these distributions gave birth to the skew family of distributions [38]. Many representations have been offered of these skewed distributions [38–41].

Asparouhov and Muthén [16] extended the canonical fundamental skew *t* distributions proposed by Lee and McLachlan [15] to structural equation models (SEM) and GMM. The skew *t* GMM was developed as an alternative to normal GMM when data are nonnormal, namely, the tails of the distribution are heavier than the normal density, and some skewness is present [15, 16]. Moreover, when fitting normal GMM, we are reducing the data to means and variances, but with skew *t* GMM we can model skewness and kurtosis as well, extracting valuable information from the data with more realistic assumptions [16].

The skew *t* family of distributions includes the skew *t* distribution and three other distributions as special cases; normal, skew normal and *t*. These four types of continuous GMM formulations are nested, thus we can directly compare fit indices and LRTs. We can, for example, compare the BIC of a *t* GMM and a skew normal, or compute LRTs to see if the fit of a skew *t* GMM is better than that of a normal GMM. As mentioned before, in the present simulation study we focus on the restricted skew *t* family of distributions. Worth mentioning is that both the restricted and unrestricted skew *t* GMMs are nested within the canonical fundamental skew *t* family of continuous GMMS as considered in Lee and McLachlan [42] and [43].

Assume we have the skew *t* distributed variable ***Y***_**i**_, following Asparouhov and Muthén [16] notation, we have

$$\begin{array}{c} Y_{i}∼\tau MST\;(\mu^{\left(g\right)},\Sigma^{\left(g\right)},\delta^{\left(g\right)},\nu^{\left(g\right)}), \end{array}$$ (5)

where *μ*^(*g*)^ is a vector of intercepts, **Σ**^(**g**)^ is a variance-covariance matrix, *δ*^(*g*)^ is a vector of skew parameters, and *ν*^(*g*)^ is a vector of degrees of freedom. Moreover, Asparouhov and Muthén [16] showed that if *δ*^(*g*)^ = 0 the skew *t* distribution reduces to a multivariate *t*; as *ν* → ∞ it reduces to the skew normal distribution; and if both *δ*^(*g*)^ = 0 and *ν* → ∞ it reduces to the normal distribution. To help understand how these distributions are all nested within the skew *t* distribution, Pyne et al. [44] use the following stochastic representation:

$$\begin{array}{c} Y_{i}=\mu^{\left(g\right)}+\delta^{\left(g\right)}\left|U_{0}\right|+U_{1}, \end{array}$$ (6)

where both ***U***_0_ and ***U***_1_ have a *t* distribution. Thus, |***U***_0_| is half a *t* distribution with a heavy right tail. The term *δ*^(*g*)^|***U***_0_| is referred as the skew factor, since it contains a vector *δ*^(*g*)^ of skewness parameters and a half *t* distribution. When *δ*^(*g*)^ is zero, then ***Y***_**i**_ has a multivariate *t* distribution. Also, as the degrees of freedom, *ν*, become larger, ***Y***_**i**_ has a multivariate normal distribution.

Model estimation for the restricted skew *t* family of distributions is achieved by the EM algorithm described by Muthén y Shedden [2] and Muthén and Asparouhov [45], and incorporating the conditional probabilities derived for this family of distributions by Asparouhov and Muthén [16].

### Goal of the study

The main aim of this simulation study is to explore the performance of fit indices (AIC, BIC and SBIC) and Likelihood Ratio Tests (VLMR-LRT, LMR adjusted LRT and BLRT) in the selection of the “true” number of latent components in GMM with multivariate continuous skew *t* family of distributions. For this purpose, normal and nonnormal data were simulated and analyzed. Nonnormal conditions of the data not considered previously in the literature are used [10, 14, 34, 35, 45, 46].

In order to achieve this goal, a single Monte Carlo simulation is conducted to explore if, in fact, fitting skew *t* GMM reduces the selection of spurious latent classes found in normal GMM [10–12, 14, 47]. The findings of this simulations study further explore the benefits of nonnormal GMM when the normally distributed random errors assumption does not hold. Moreover, simulation results were corroborated with a real data application example.

This simulation study extends those performed by Bauer and Curran [10, 12] and Guerra-Peña and Steinley [14] regarding classification problems of normal GMM, to the skew *t* family of distributions, which includes normal, skew normal, *t* and skew *t*.

The main hypothesis of this simulation study is that analysis of data with skew and kurtosis using GMM based on skewed distributions (e. g., skew normal or skew *t*) will yield results that select the “true” number of latent classes more often in comparison with GMM based on symmetric distributions (e. g., normal and *t*). Namely, the likelihood of these skew GMM will be a better representation of the data, and spurious classes to compensate for asymmetry or heavy tails of the data will not be necessary.

## Materials and methods

### Simulation design

**Fig 1:**
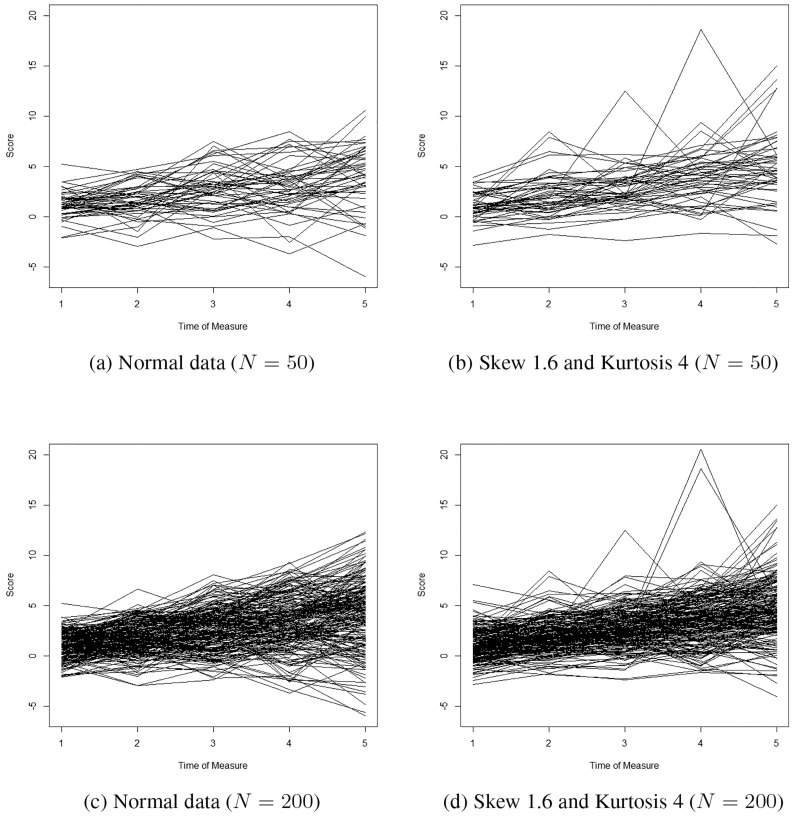
Line plots for normal (skew 0 and kurtosis 0) versus nonnormal data (skew 1.6 and kurtosis 4).

Data were generated using the linear growth model of Bauer and Curran [10]. Only one group exists in the simulated data. Five occasions of measurement were simulated. In this model, mean trajectory increases over time (*μ*_*α*_ = 1.00 and *μ*_*β*_ = 0.80). Both intercepts and slopes were allowed to vary among individuals (Ψ_*α*_ = 1.00 and Ψ_*β*_ = 0.20). Intercepts and slopes were designed to have a slight positive correlation (Ψ_*αβ*_ = 0.11). Moreover, error variances for the repeated measures are set to increase over time (**Θ**_**ϵ**_ = *diag*[1.00;1.42;2.25;3.47;5.09]). Three distributional conditions were explored: normal (skew = 0 and kurtosis = 0), slightly nonnormal (skew = 1 and kurtosis = 2), and nonnormal (skew = 1.6 and kurtosis = 4). Conditions that were not exactly normal were generated and then transformed to the specific values of skew and kurtosis using the Vale and Mauerlli [48] extension to the Fleishman [49] method as implemented in EQS 6.3 [46]. Fig 1 presents some examples of the normal and nonnormal simulated data. These nonnormal conditions are often found in data in the social sciences [13] and would not be of any concern to an applied researcher fitting normal GMM. Moreover, in addition to the distributional conditions, four sample size conditions were simulated (*N* = 50, *N* = 200, *N* = 800 and *N* = 3, 200) and 500 samples were generated for each condition.

The aforementioned simulation conditions result in a total of 6,000 data sets that were generated and analyzed with all four of the distributions included in the skew *t* family of continuous GMM. Furthermore, one- and two-class models were fitted to the data. The EM algorithm with the MLR option and robust standard errors were used via Mplus 7.4 [17]. Parameters used to simulate the data were used as starting values and one hundred random starts were specified to avoid local optimal solutions and non-convergence issues according to recommendations in the literature [23–25]. Two group models were given starting values with a low and a high group (${\hat{\mu }}_{\alpha }=0.00$ and ${\hat{\mu }}_{\beta }=0.00$ for class 1, and ${\hat{\mu }}_{\alpha }=1.50$ and ${\hat{\mu }}_{\beta }=1.60$ for class 2) consistent with other simulation studies [10, 27, 48]. Moreover, models were given 1, 000 iterations to converge and convergence rates and time to converge were recorded for each model. These convergence rates and convergence times are presented in Tables 1 and 2, respectively.

**Table 1:** Convergence rate by distribution, data condition and sample size (500 replications).

N	Distribution	Normal	Skew Normal	Skew *t*	*t*
50	Normal	492 (0.98)	317 (0.63)	433 (0.87)	35 (0.07)
	S. Nonnormal	484 (0.97)	348 (0.70)	417 (0.83)	51 (0.10)
	Nonnormal	485 (0.97)	359 (0.72)	406 (0.81)	56 (0.11)
200	Normal	488 (0.98)	286 (0.57)	450 (0.90)	46 (0.09)
	S. Nonnormal	498 (0.96)	288 (0.58)	432 (0.87)	98 (0.20)
	Nonnormal	481 (0.96)	274 (0.55)	432 (0.86)	125 (0.25)
800	Normal	488 (0.98)	281 (0.56)	470 (0.94)	116 (0.23)
	S. Nonnormal	483 (0.97)	276 (0.55)	459 (0.92)	149 (0.30)
	Nonnormal	491 (0.98)	264 (0.53)	464 (0.93)	178 (0.36)
3,200	Normal	470 (0.94)	295 (0.59)	473 (0.95)	217 (0.43)
	S. Nonnormal	500 (1.00)	457 (0.91)	489 (0.98)	185 (0.37)
	Nonnormal	500 (1.00)	445 (0.89)	484 (0.97)	230 (0.46)

Values are frecuencies (and proportions) of convergence across replications. Each replication was allowed 1,000 iterations to converge. Data conditions are normal (s = k = 0), s. nonnormal (slightly nonnormal, s = 1, k = 2) and nonnormal (s = 1.6, k = 4).

**Table 2:** Time of convergence by distribution and sample size for 1-class and 2-class solutions (500 replications).

N	Classes	Distribution	Normal	Skew Normal	Skew *t*	*t*
50	1	Normal	0.43	0.27	0.45	0.18
		S. Nonnormal	0.63	0.07	0.27	0.12
		Nonnormal	0.45	0.57	0.25	0.12
	2	Normal	191.63	12.58	12.83	7.18
		S. Nonnormal	43.68	1.30	9.00	2.90
		Nonnormal	39.03	1.32	8.57	2.83
200	1	Normal	0.50	0.25	1.40	0.37
		S. Nonnormal	0.83	0.08	0.72	0.27
		Nonnormal	0.53	0.70	0.67	0.25
	2	Normal	35.23	0.25	1.40	0.37
		S. Nonnormal	0.83	0.08	0.72	0.27
		Nonnormal	41.93	2.22	24.23	8.03
800	1	Normal	3.20	0.42	4.97	1.18
		S. Nonnormal	1.07	0.17	2.38	0.83
		Nonnormal	0.63	1.57	2.47	0.85
	2	Normal	109.48	8.13	215.70	46.67
		S. Nonnormal	68.20	5.77	80.90	23.13
		Nonnormal	88.27	5.77	84.70	29.58
3,200	1	Normal	5.92	1.03	18.35	4.30
		S. Nonnormal	1.58	0.47	8.63	3.15
		Nonnormal	0.88	4.42	10.25	3.15
	2	Normal	271.03	25.08	631.22	148.68
		S. Nonnormal	123.73	20.23	319.00	97.63
		Nonnormal	59.57	21.00	334.02	122.53

Values are a range of minutes that the EM algorithm needed to converge for all replications. Each replication was allowed 1,000 iterations to converge.

Type I error (false positive) rates were computed as the proportion of times fit statistics and LRT favored the incorrect, two-class solution. In the case of fit statistics, the preferred model was the one with the smallest value. Test statistics comparing the k-class with the k-1-class solution were used for LRTs.

The selection accuracy of these fit indices, in similar conditions of nonnormal data, has been analyzed for normal GMM [10, 14], as well as for LRT [14]. Nevertheless, in the case of the skew *t* family of distributions GMM, the efficiency of fit indices and LRT in selecting the accurate number of latent classes has not been exhaustively explored in the literature. Since these members of the family of distributions are nested with each other, direct comparison of fit indices and LRT are possible [16].

### Educational data example

We used the Early Childhood Longitudinal Study-Kindergarten (ECLS-K) class database [50]. These data are an example of skewed variables that are commonly modeled through normal GMM. More specifically, we used four time points of reading achievement collected between 1998 and 2000 panels of ECLS-K. Measurement correspond to children assessment in both the fall and spring of kindergarten and first grade. Furthermore, these assessments represent reading skill improvement over 18 months; note the measurement are not equidistant. The reading achievement data used here were initially pulled from Kaplan [51]. For the purpose of this example, we removed children with missing data and were left with a total sample size of 3856 children.

**Fig 2:**
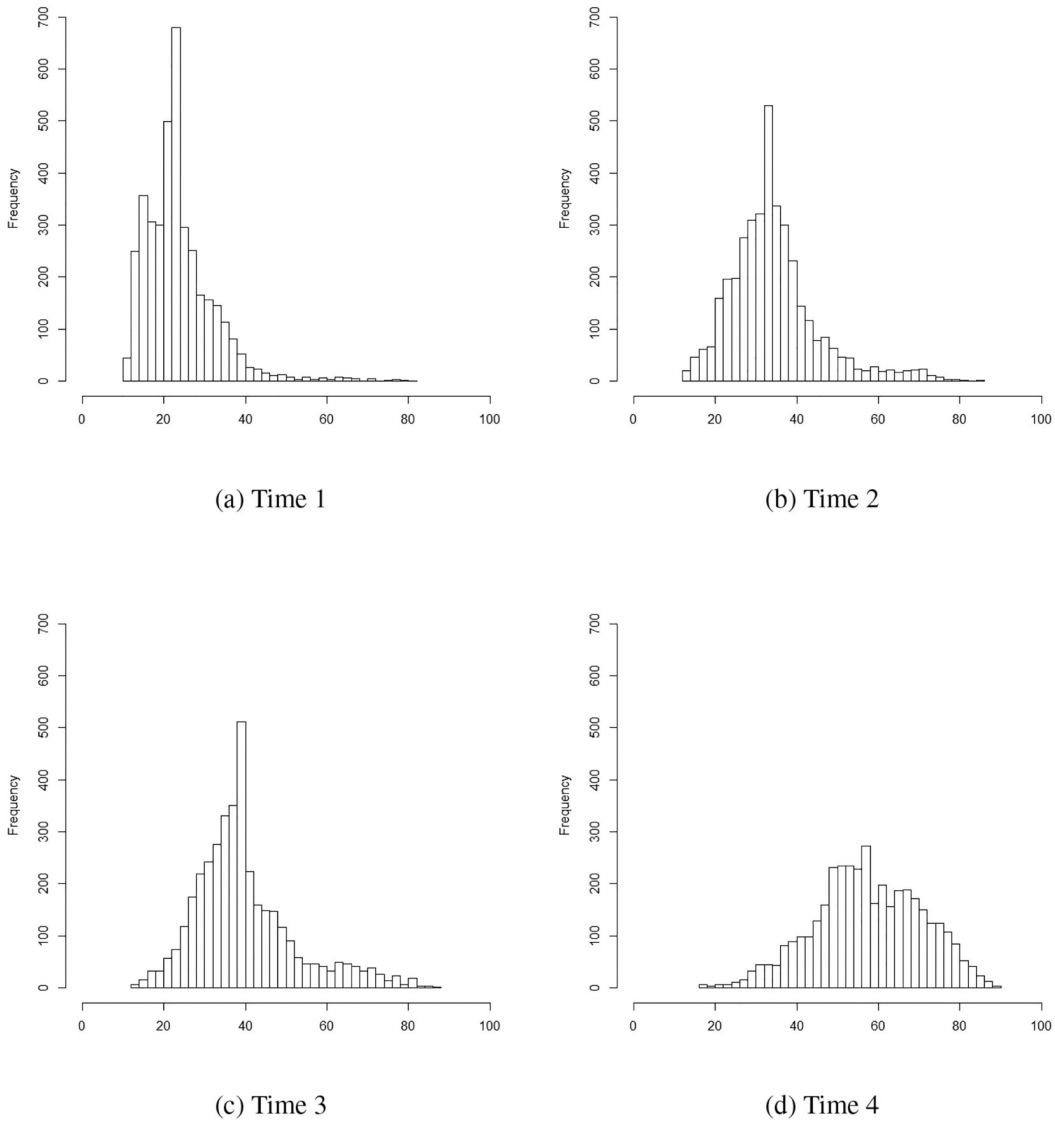
Histogram for reading scores for each time point.

Descriptive statistics and correlation matrix of reading scores for the ECLS-K data are presented in Table 3. Skewness and kurtosis are present in all four time points; values are higher for the first assessment of reading, and diminish across time points. The distributions of reading achievement at each of the four time points are provided in Fig 2 (plots a–d). We analyzed the ECLS-K data with the skew *t* family of continuous GMM with both linear and quadratic slopes and the heterogeneous variance, and using the autoregressive variance-covariance matrix structure presented by McNeish and Harring [52]. Likelihood ratio test results are not presented since these are not available in Mplus when using non-linear constrains.

**Table 3:** Descriptive statistics and correlation matrix of reading scores for each of the four time points for ECLS-K database.

Time	Mean	Variance	Skewness	Kurtosis	time 1	time 2	time 3
1	23.90	78.91	**1.97**	**6.86**			
2	34.43	120.18	**1.22**	**2.37**	**0.81**		
3	39.48	154.78	**1.05**	**1.26**	**0.77**	**0.89**	
4	57.40	172.51	**-0.15**	**-0.36**	**0.64**	**0.74**	**0.80**

*N* = 3856. Significant coefficients appear bolded.

## Results

Table 1 shows the convergence rates of the simulated data by sample size and GMM underlying distribution. Normal GMM showed the highest convergence rates with the minimum value reported for a sample size of 200, with a range of 92.8 to 98.4. On the other hand, the *t* distributed GMM showed the lowest convergence rates with a minimum value of 7.0 to 11.2 for a sample size of 50, and a maximum value of 19.4 to 68.8 for a sample size of 800. The time it took the EM algorithm to find a solution across the 500 data sets for each simulation condition is reported in Table 2. Larger sample sizes were associated with longer computation time across the four GMM distributions considered in this study. Moreover, since only one latent class was simulated, the 2-class model (wrong model) took considerably longer than the 1-class model across sample sizes and GMM distributions. The skew *t* GMM showed to be the most computationally intensive of all distributions considered, with the 2-class model for a sample size of 3,200 taking as long as 318.63 to 631.22 minutes to run all 500 samples. Both skew normal and *t* GMM, that had the lowest convergence rates (see Table 1), also converged the fastest. This might be due to the limit of 1,000 iterations to converge and, if convergence was not achieved, the replication would be considered as that it did not converge.

Tables 4–6 show false positive rates for fit indices. Note that only one latent group exists in the simulated data, hence these values represent the times the applied researcher would select the wrong model by making a decision based on these fit indices. In these Tables we can see that the three fit indices used in this study, in general, had higher false positive rates for normal GMM, than for the skew normal, skew *t* and *t* GMM, except for the BIC false positive rate when sample size was 3,200 and the simulated data was normal (see Table 4). This trend of the BIC outperforming the AIC and SBIC holds true across the skew *t* family GMM and distributional conditions of the data, becoming more apparent as sample size increases.

**Table 4:** Fit index and LRT false positive rate (of 500 samples) for all models and normal data (skew and kurtosis = 0).

*N*	Distribution	AIC	BIC	SBIC	VLMR-LRT	LMR-LRT	BLRT
50	Normal	0.81	0.35	0.99	0.13	0.11	**0.03**
	Skew Normal	0.08	**0.00**	0.53	0.12	0.10	
	Skew *t*	**0.01**	**0.00**	0.06	**0.05**	**0.05**	
	*t*	0.09	**0.00**	0.83	**0.03**	**0.03**	
200	Normal	0.73	0.09	0.67	0.11	0.10	**0.03**
	Skew Normal	**0.04**	**0.00**	**0.03**	0.12	0.12	
	Skew *t*	**0.00**	**0.00**	**0.00**	0.11	0.11	
	*t*	**0.00**	**0.00**	**0.00**	**0.00**	**0.00**	
800	Normal	0.72	**0.03**	0.27	0.17	0.15	**0.03**
	Skew Normal	**0.02**	**0.00**	**0.00**	0.09	0.08	
	Skew *t*	**0.00**	**0.00**	**0.00**	**0.03**	**0.03**	
	*t*	**0.05**	**0.00**	**0.00**	**0.02**	**0.02**	
3,200	Normal	0.81	**0.00**	0.08	0.23	0.22	**0.03**
	Skew Normal	**0.03**	**0.00**	**0.00**	**0.03**	**0.03**	
	Skew *t*	**0.00**	**0.00**	**0.00**	**0.00**	**0.00**	
	*t*	0.06	**0.00**	**0.00**	**0.03**	**0.03**	

AIC = Akaike’s information criterion; BIC = Bayesian information criterion; SBIC = Sample corrected BIC; VLMR-LRT = Voung-Lo-Mendell-Rubin LRT; LMR-adjusted LRT = Lo-Mendell-Rubin adjusted LRT; and BLRT = Bootstrap LRT. Type I error rates ≤ 0.05 appear bolded.

**Table 5:** Fit index false positive rate (of 500 samples) for all models and slightly nonnormal data (skew = 1 and kurtosis = 2).

*N*	Distribution	AIC	BIC	SBIC	VLMR-LRT	LMR-LRT	BLRT
50	Normal	0.77	0.68	0.99	0.18	0.16	**0.03**
	Skew Normal	0.08	**0.01**	0.58	0.10	0.10	
	Skew *t*	**0.00**	**0.00**	**0.04**	**0.02**	**0.02**	
	*t*	0.29	**0.00**	0.78	0.16	0.16	
200	Normal	0.76	0.09	0.71	0.17	0.17	**0.02**
	Skew Normal	0.15	**0.00**	0.12	0.15	0.14	
	Skew *t*	**0.00**	**0.00**	**0.00**	0.06	0.06	
	*t*	0.36	**0.01**	0.34	0.27	0.24	
800	Normal	0.95	0.15	0.69	0.46	0.43	0.14
	Skew Normal	0.45	**0.00**	0.15	0.35	0.34	
	Skew *t*	**0.00**	**0.00**	**0.00**	**0.01**	**0.01**	
	*t*	0.87	**0.02**	0.46	0.48	0.48	
3,200	Normal	1.00	0.86	0.99	0.87	0.87	0.94
	Skew Normal	0.96	0.27	0.67	0.75	0.74	
	Skew *t*	**0.00**	**0.00**	**0.00**	**0.01**	**0.01**	
	*t*	1.00	0.99	1.00	0.87	0.87	

AIC = Akaike’s information criterion; BIC = Bayesian information criterion; SBIC = Sample corrected BIC; VLMR-LRT = Voung-Lo-Mendell-Rubin LRT; LMR-adjusted LRT = Lo-Mendell-Rubin adjusted LRT; and BLRT = Bootstrap LRT. Type I error rates ≤ 0.05 appear bolded.

**Table 6:** Fit index false positive rate (of 500 samples) for all models and nonnormal data (skew = 1.6 and kurtosis = 4).

*N*	Distribution	AIC	BIC	SBIC	VLMR-LRT	LMR-LRT	BLRT
50	Normal	0.81	0.32	0.99	0.16	0.14	**0.02**
	Skew Normal	0.09	**0.00**	0.60	0.09	0.09	
	Skew *t*	**0.01**	**0.00**	0.06	**0.04**	**0.04**	
	*t*	0.27	**0.00**	0.86	0.18	0.18	
200	Normal	0.83	0.15	0.79	0.22	0.21	**0.04**
	Skew Normal	0.23	**0.00**	0.20	0.15	0.15	
	Skew *t*	**0.01**	**0.00**	**0.00**	0.10	0.10	
	*t*	0.49	**0.01**	0.44	0.31	0.30	
800	Normal	1.00	0.41	0.88	0.63	0.62	0.41
	Skew Normal	0.57	**0.01**	0.25	0.36	0.36	
	Skew *t*	**0.00**	**0.00**	**0.00**	**0.03**	**0.03**	
	*t*	0.99	0.17	0.80	0.64	0.63	
3,200	Normal	1.00	1.00	1.00	0.92	0.91	1.00
	Skew Normal	0.99	0.47	0.82	0.75	0.74	
	Skew *t*	**0.00**	**0.00**	**0.00**	**0.01**	**0.01**	
	*t*	1.00	0.99	1.00	0.87	0.87	

AIC = Akaike’s information criterion; BIC = Bayesian information criterion; SBIC = Sample corrected BIC; VLMR-LRT = Voung-Lo-Mendell-Rubin LRT; LMR-adjusted LRT = Lo-Mendell-Rubin adjusted LRT; and BLRT = Bootstrap LRT. Type I error rates ≤ 0.05 appear bolded.

In Tables 4–6 we can also see that, in the case of the normal GMM, and slightly nonnormal and nonnormal data conditions (skew = 1 and kurtosis = 2, and skew = 1.6 and kurtosis = 4, respectively), BIC type I error rates are much higher than the maximum accepted for this study (0.05). These BIC values range from 0.09, for a sample size of 200 and the skew of 1 and kurtosis of 2 data condition, to 1.00, the skew of 1.6 and kurtosis of 4 data condition and a sample size of 3,200. The latter result means that for all converged replications, the BIC for the “false” 2-class model was smaller (better) than the BIC for the “true” 1-class model. Note that for sample sizes of 3,200 the BIC performed as poorly as the SBIC and the AIC, favoring the 2-class solution from 86 to 100% of the replications. These results show that the BIC is not as useful for identifying the correct number of latent components in normal GMM.

Skew normal, *t* and skew *t* GMMs have better (smaller) overall false positive rates than normal GMM, across sample size and data distributional conditions (see Tables 4–6). Nevertheless, the skew *t* GMM presented the smallest type I error rates for all fit statistic across sample sizes and distributional conditions. Moreover, as sample size increased and the data became more nonnormal, fit statistic for the skew *t* GMM presented progressively smaller false positive rates and the normal, skew normal and *t* GMMs progressively larger type I error rates (e.g., values in parenthesis as high as 1.00).

**Fig 3:**
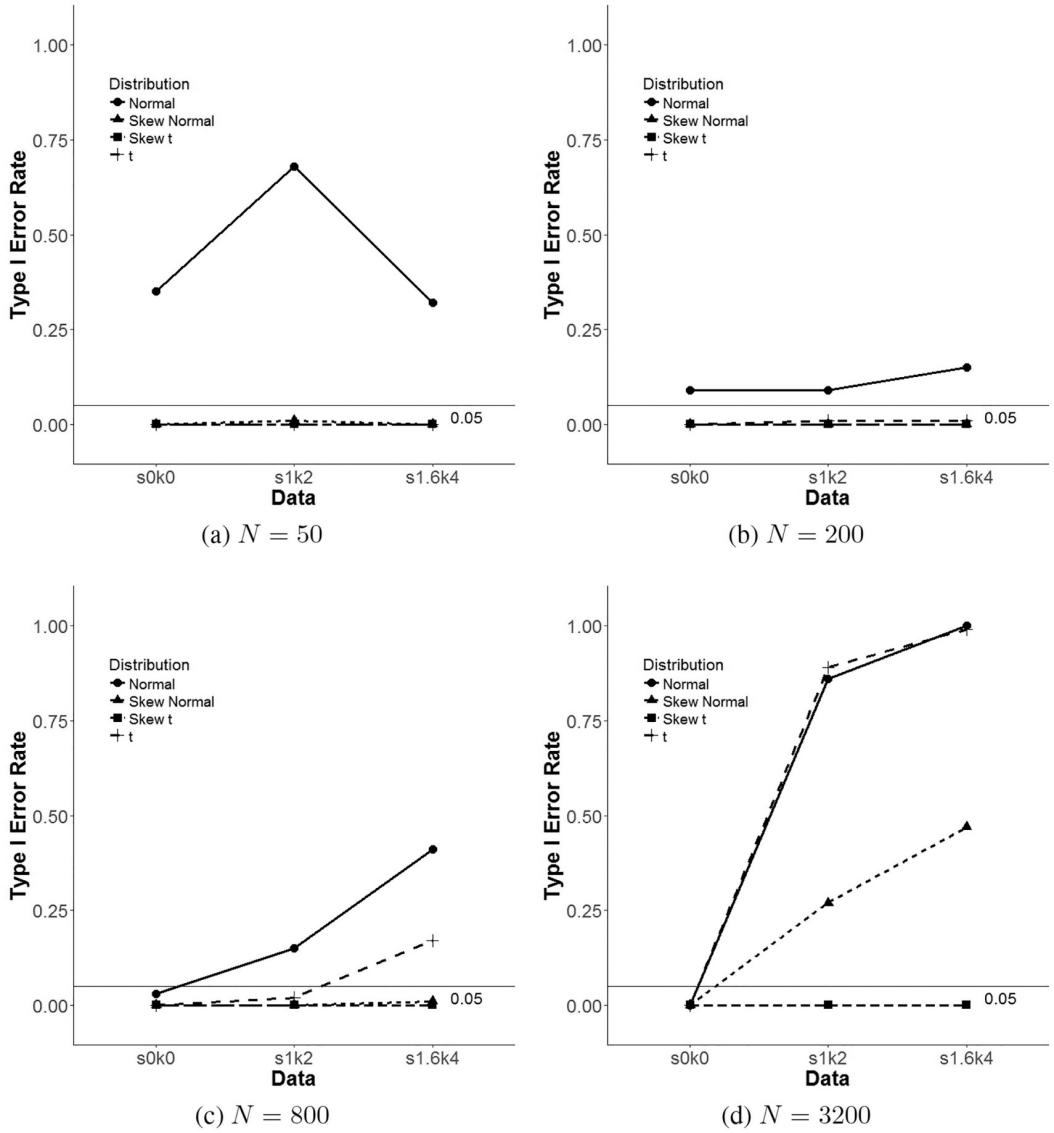
Line plots for BIC false positive rate across skew-*t* family GMM for each distribution condition.

Fig 3 presents line plots comparing BIC false positive rates for all four distributions of the skew *t* family GMM. The BIC was used since it has been shown to outperform other fit indices both in this study and in the literature [14, 16, 33, 34, 48]. The four lines in Fig 3a–3d represent these four members of the skew *t* family of continuous distributions. Furthermore, false positive rates are shown in the *y*-axis and the three distributional conditions (e.g., normal, s. nonnormal & nonnormal) in the *x*-axis. Each figure also has a reference line at the 0.05 type I error rate mark to indicate the acceptable level. Namely, only those GMMs with values below this reference line are considered to have acceptable type I error rates. The only different simulation condition between Fig 3a–3d is sample size, that went from *N* = 50 to *N* = 3, 200, respectively. Fig 3a and 3b show that for small sample sizes (e.g., *N* = 50 and *N* = 200) normal GMM has higher type I error rates and the skew normal, skew *t* and *t*, and these three GMMs performed equally well with false positive rates below 0.05. This changed for larger samples sizes (e.g., *N* = 800 and *N* = 3, 200) as sample size increased, first *t* GMM for the nonnormal condition (skew = 1.6 and kurtosis = 4), and then both the *t* GMM and the skew normal GMM for the slightly nonnormal (skew = 1 and kurtosis = 2) and nonnormal conditions, showed type I error rates much larger than the skew *t* GMM. Tables 4–6 and Fig 3a–3d show that for the four GMMs considered in this simulation study, only the skew *t* GMM achieved appropriate type I error rates regardless of sample size and nonnormality of the data. It is important to note that for the normal data condition (skew = 0 and kurtosis = 0) and with respect to the BIC, all considered models showed false positive rates smaller than 0.05, except the normal GMM.

Asparouhov and Muthén [16] showed that GMMs for the skew *t* family of continuous distributions are nested. Hence, Table 7 shows average BIC scores across all replications for all simulation conditions. Smaller BIC values for 1-class versus 2 classes solutions for each distribution of GMM is presented in bold numbers and the smallest overall BIC values per sample skewness and kurtosis condition is presented with the superscript *a*. This was done with the purpose of exploring whether the best relative model, selected by the BIC, would correspond with the smallest type I error rate among considered models. These results would give an example of the conclusions applied researchers might come to when only considering fit indices in the selection of the number of latent classes in GMM. Table 7 shows that for the normal data condition (skew = 0 and kurtosis = 0) the smallest BIC average value corresponds to the 1-class solution for the normal GMM. This is an interesting result since only one class exists in the simulated data and because we have seen from Tables 4–6 and Fig 3 that the normal GMM showed the highest type I error rate across simulation conditions. Notice that results presented in Tables 4–6 and Fig 3 compared fit indices values within the same member distribution of the skew *t* GMM family, and Table 7 compares BIC scores across all these member distributions. BIC scores for nonnormal distributed data conditions were smaller for the 1-class solution *t* distributed GMM. Hence, when skew and kurtosis were present in the data, the *t* GMM shows a better relative fit compared to normal, skew normal and skew *t* GMM. These results would direct a researcher onto fitting *t* distributed GMM, which is symmetric, to nonnormal data, even though the simulated data was skewed, and type I error rates and convergence rates, related to this parametrization of GMMs, have been shown in this simulation study to be problematic.

**Table 7:** BIC of one-class versus two-class models (of 500 samples) for all models by sample size and distributional conditions.

*N*	Distribution	Skew 0, kurtosis 0		Skew 1, kurtosis 2		Skew 1.6, kurtosis 4	
		1 class	2 classes	1 class	2 classes	1 class	2 classes
50	Normal	**1045.66**^a^	1047.53	**1039.19**	1041.20	**1037.15**	1038.70
	Skew Normal	**1053.10**	1073.66	**1045.92**	1065.84	**1043.55**	1065.08
	Skew *t*	**1056.11**	1085.08	**1042.13**	1070.92	**1035.99**	1065.16
	*t*	**1049.38**	1070.90	**1035.41**^a^	1050.77	**1029.96**^a^	1044.12
200	Normal	**4106.44**^a^	4117.22	**4098.78**	4110.34	**4096.17**	4106.24
	Skew Normal	**4115.58**	4143.18	**4106.28**	4128.64	**4102.38**	4128.73
	Skew *t*	**4119.77**	4163.27	**4070.72**	4111.92	**4046.34**	4085.75
	*t*	**4111.41**	4136.17	**4063.40**^a^	4079.85	**4040.64**^a^	4058.50
800	Normal	**16309.73**^a^	16328.13	**16294.78**	16304.96	**16290.77**	16292.09
	Skew Normal	**16321.31**	16362.82	**16298.96**	16326.21	**16286.60**	16304.20
	Skew *t*	**16327.59**	16381.07	**16135.26**	16186.08	**16037.69**^a^	16088.54
	*t*	**16316.36**	16344.86	**16129.83**^a^	16138.73	**16038.87**	16039.22
3,200	Normal	**65068.47**^a^	65091.01	65064.35	**65042.46**	65063.69	**64997.60**
	Skew Normal	**65082.94**	65142.69	**65048.89**	65057.66	65012.48	**65009.81**
	Skew *t*	**65089.90**	65157.76	**64342.32**	64407.58	**63953.73**	64018.50
	*t*	**65076.08**	65117.83	64352.65	**64315.98**^a^	63990.71	**63935.55**^a^

BIC = Bayesian information criterion. The lowest BIC values within the same distribution appear bolded.

^a^Lowest BIC values across classes and distributions.

Another interesting result shown in Table 7 is that for nonnormal data and sample sizes of 50, 200 and 800 the best models (smallest BIC) were 1-class solutions. On the other hand, when the sample size was large (e.g., *N* = 3, 200) the better fitting model was the 2-class *t* distributed GMM. Moreover, for this large sample size only skewed GMM selected the correct 1-class solution, and in the distributional condition of a skew of 1.6 and kurtosis of 4, only the skew *t* GMM favored the “true” number of latent classes. Hence, for large sample sizes and nonnormal data the BIC for the normal, skew normal and *t* GMM favors solutions with more latent classes than the model used to simulate the data, with the exception of the skew *t* GMM.

**Fig 4:**
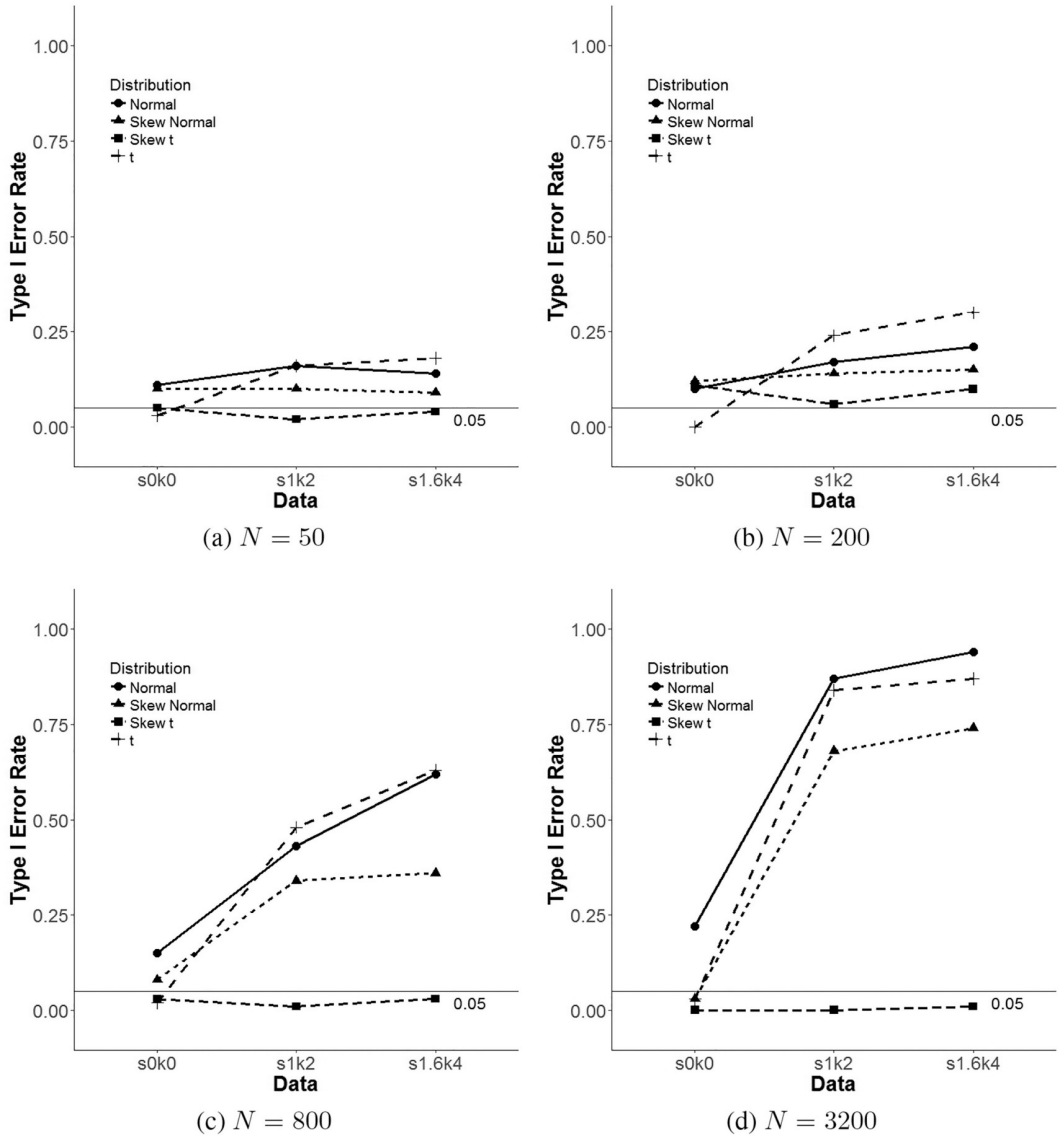
Line plots for LMR-adjusted LRT false positive rate across skew-*t* family GMM for each distribution condition.

Results for LRT are shown in the right-hand side of Tables 4–6 and Fig 4. In the same way as fit indices were presented in the left-hand side of these same tables, these tables show the proportion of times LRTs selected the model with the “false” 2-class solution within the particular skew *t* family GMM, and across distributions of the simulated data and sample sizes. VLMR-LRT and LMR-adjusted LRT type I error rates are presented for all members of the skew *t* family GMM. Nevertheless, BLRT false positive rate results are reported for only the normal GMM since this LRT is not yet available for other members of this family of distributions on Mplus 7.4 [17], the statistical packages used for this simulation study.

For the normal GMM, the BLRT is the only LRT with acceptable levels of type I error rates for small sample sizes (e.g., *N* = 50 and *N* = 200) ranging from 0.02 to 0.04 across skew and kurtosis values. Nonetheless, for larger sample sizes (e.g., *N* = 800 and *N* = 3, 200) only the normal data condition presents this pattern of results. BLRT type I error rates for the s. nonnormal and nonnormal data conditions are much larger than the cutoff value of 0.05, ranging from 0.14 to 1.00. Namely, for a sample size of 3,200 and a skew of 1.6 and a kurtosis of 4, the BLRT selected the incorrect 2-class model 100% of the replications that converged. Also, with respect to normal GMM, the VLMR and LMR-adjusted LRTs show false positive rates larger than the 0.05 reference value for normal and nonnormal data conditions. The VLMR-LRT presented type one error rates ranging from 0.00 to 0.92, in Tables 4–6. The LMR-adjusted LRT showed almost identical results with slightly smaller type I error rates in some cases.

Fig 4 shows that as sample size increases and the data becomes more nonnormal, only the skew *t* GMM shows type I error rates of LRTs that are under 0.05, and that GMMs based on the other members of the family show false positive rates that become increasingly worse. This Fig shows that not only does the skew *t* present the smallest type one error rates, but it is also the only GMM that falls under the 0.05 cutoff value across sample sizes and distributional conditions of the data. Fig 4a and 4b show that the skew *t* GMM performs several times better than GMMs based on the other three distribution considered for small sample sizes and we can see in Fig 4c and 4d how the skew *t* error rate falls to 0.00 and the others approach 1.00 as sample size, skewness, and kurtosis increases.

In order to corroborate the simulation results presented above, we analyzed the ECLS-K data with all members of the skew *t* family of continuous GMM’s. BIC scores for the normal, skewed normal, *t* and skewed *t* GMM for these data are presented in Table 8. Results are consistent with our simulation findings. The skew *t* GMM selected fewer classes than any other member of the skew *t* family, with four classes. The normal GMM was the second most conservative, suggesting that the five class solution is best. Moreover, both the skew normal and the *t* GMM’s favored the six classes solution.

**Table 8:** Number of classes suggested by the BIC for each member of the skew t GMM family for the ECLS-K database.

Number of classes	Normal	Skew Normal	Skew *t*	*t*
1	107856.48	107598.56	107312.89	107306.81
2	105566.12	104717.86	104699.88	105555.77
3	104831.19	104233.29	104183.99	104854.35
4	104268.16	103848.48	**103995.84**	104285.40
5	**104121.07**	103793.69	104100.77	104151.61
6	104323.23	**103790.16**^a^		**104077.56**
7		104028.70		104200.93

BIC = Bayesian information criterion. The lowest BIC values within the same distribution appear bolded.

^a^Lowest BIC values across classes and distributions.

## Discussion

As the use of GMMs continues to increase in the applied literature, the need for methods that detect the “true” number of latent classes will also increase. Applied researchers rely on measures of fit to aid in assessing optimal latent class solutions, and then these final models are substantively interpreted. This makes the detection of the acurate selection of latent classes of utmost importance. The current study aimed to assess alternative modeling techniques for detecting “true” classes within GMMs. Specifically, the restricted skew *t* family of continuous GMMs as presented by Asparouhov and Muthén [16] was examined over simulation conditions and using fit indices not considered previously. It also includes results of the effectiveness of LRTs in the selection of the number of latent classes within nonnormal GMMs.

Type I error rates for the use of fit statistics and LRTs in all four of the GMMs based on the skew *t* family of continuous distributions were reported. This family of distributions includes GMMs based on the normal, the skew normal, the *t* and the skew *t* distributions. Simulation results show that only the skew *t* GMM had false positive rates, for fit indices and LRT, below the 0.05 cutoff value, across sample sizes, and different levels of data nonnormality. These results are consistent with Asparouhov and Muthén [16] as the authors suggest that using the skew *t* GMM might reduce the overextraction of spurious classes in GMM. The *t* distributed GMM works well for normal data (e.g., skew = 0 and kurtosis = 0), but this is hardly helpful since data in the social sciences are seldom normal [13]. Nevertheless, as skewness and kurtosis are introduced into the data and sample size increases, so does the type I error rate of the fit indices and LRTs of the *t* distributed GMM.

Previous results on the problems of fitting normal GMM were confirmed in the current research; the overextraction of latent classes in the case of nonnormal data by the use of fit indices [10–12, 14], and by the use of LRTs [14]. Namely, for data with skewness and kurtosis, the one-class solution for the normal GMM is a poor representation of the data and spurious classes are necessary for model fit improvement. The skew *t* GMM results did not select solutions with spurious classes in the simulation conditions explored, thus it shows promise in ameliorating some of the issues with GMM first exposed by Bauer and Curran [10–12].

The BIC outperformed the AIC and SBIC across the skew *t* family GMM and distributional conditions of the data, becoming more apparent as sample size increased. These results are consistent with previous research that explored the efficiency of fit indices in the case of normal GMM [14, 33, 34, 53]. Nevertheless, the results from this simulation study also show that the BIC is not as useful for identifying the correct number of latent components in normal GMM as previously suggested in large sample sizes and nonnormal populations [33, 34, 53].

These results could serve as guides to researchers in the social sciences that wish to run GMM on real world data. These researchers could use the tables and figures presented here to be aware of the risks selecting latent classes that do not exist in the data. Applied researchers could use these results as a guide for the decision of which formulation of GMM, within the skew *t* family of distributions, to use in order to avoid the selection of “false” latent classes. Given that real life data are not normal, these results suggest that the skew *t* GMM should be used rather than the normal, skew normal, and *t* distributed GMM.

Further research is needed to determine other important considerations for the use of GMM based on the skew *t* family of continuous distributions, such as type II error (false negative rates), parameter bias and effect an incorrect specification of the functional form of the growth process. It would also be interesting to compare the performance of these frequentists GMMs with Bayesian GMMs as those explored by Depaoli [54].

These results are consistent with Asparouhov and Muthén [16] as the authors suggest that using the skew *t* GMM might reduce the overextraction of spurious classes in GMM. It is important to note that for the normal data condition (skew = 0 and kurtosis = 0) and with respect to the BIC, all considered models showed false positive rates smaller than 0.05.

This study has several limitations. Only type I error rates were of interest and for this purpose, only one-class solutions were simulated, thus considered correct. When a particular parametrization of GMM led to the two-latent class solution, it was considered a false positive error. This is a very specific example of type I errors and other conditions need to be explored. Depaoli, Winter, Lai and Guerra-Peña [54] have recently publish a paper that addresses this very issue. Another limitation is that only the restricted skew *t* family of continuous GMM was considered. The current study needs to be extended to other forms of nonnormal GMMs. Moreover, future research is needed to explore type II error rates (false negative rates) and parameter estimation bias should also be investigated to make sure that this and other nonnormal GMMs are free of the limitations of normal GMMs.
